# The Knowledge Content of the Greek Production Structure in the Aftermath of the Greek Crisis

**DOI:** 10.1007/s13132-022-01095-7

**Published:** 2023-01-31

**Authors:** Anna-Maria Kanzola

**Affiliations:** National and Kapodistrian University of Athens, Athens, Greece

**Keywords:** Production structure, Knowledge content, Human capital, Greece

## Abstract

Contrary to the traditional approaches for the evaluation of human capital, this study proposes the assessment of human capital through its qualitative characteristics (knowledge, skills, abilities, and working activities). To investigate the knowledge content density in the Greek production structure for the year 2018, the methodology followed concerns a sectoral analysis of the economy and the matching of each occupation with four knowledge items from the O*NET database. The findings indicate that the prevailing knowledge characteristics (66% of total) of the Greek production structure of 2018 concern a simple rather than sophisticated knowledge background which is reflected in the economic structure and in middle and low specialization of the workforce. This study contributes to the literature by providing an original and robust way to evaluate the knowledge content in total within the economy without being based on model forecasts but sectoral data. Furthermore, it provides policymaking insights for the intervention in specific areas of the knowledge content which could “nudge” economic growth of the existing production structure. The grounds for policymaking concern education, reskilling, and especially lifelong learning programs.

**Supplementary Information:**

The online version contains supplementary material available at 10.1007/s13132-022-01095-7.

## Introduction

Economic structures are the fundamental cause of economic performance because the free market cannot ensure growth-enhancing transformations (Constantine, 2017). Given that human capital quality is a determinant factor of research and development (Romer, 1990), there is a reciprocal relationship between human capital and economic growth (Mincer, 1996). Therefore, it is safe to assume that the quality and accumulation of human capital determine an economy’s productive capacity.

Traditionally, the evaluation of human capital is based on quantitative criteria such as the number of years of training, graduation percentages from secondary and higher education, the percentage of people who choose to lifelong trained, and generally the evaluation of the returns on investment spending (Kwon, 2009; Mincer, 1958, 1974, 1978; Nehru et al., 1993; Psacharopoulos, 1985; Psacharopoulos & Patrinos, 2018). Such approaches account for the positive externalities produced by the accumulation of human capital ignoring the structural characteristics of human capital, a fact which has resulted in criticism (El Kadiri Boutchich, 2019; Petrakis & Stamatakis, 2002).

On the other hand, qualitative methods evaluate the quality of human capital by reference to different concepts such as knowledge and skills management (El Kadiri Boutchich, 2019). The evaluation of human capital through its qualitative characteristics is relatively recent and it refers to the task approach to the labor markets (Acemoglu & Autor, 2011; Autor, 2013) which states that the fundamental units of production are combined job tasks to produce outcomes. Following the O*NET database^1^ categorization, the features embodied in human capital are (a) knowledge, (b) skills, (c) abilities, and (d) working activities.

The present study consists of a qualitative evaluation of human capital regarding its knowledge component. The proposed methodology combines the co-examination of the production structure and the knowledge required for the performance of tasks by an occupation. Eventually, this approach brings into light a quantified description of the knowledge characteristics of the economy as a total. Such a description of the knowledge content has only been attempted inside the firm (Andreeva & Kianto, 2011). Hence, the present study significantly contributes to the existing literature by implementing an explicit, direct, and reliable way for the measurement of human capital in the economy by means of occupational data. This method is reliable because it is based on employment data, not statistical forecasts and model measurements.

The country of reference is Greece for the year 2018 for which there are available data from the Hellenic Statistical Authority. Methodologically, this study employs a sectoral analysis using data (unpublished) from the Workforce Study of the Hellenic Statistical Authority. Specifically, the methodology is based on matching each occupation in the economy with the sector within it operates. Thus, all the workers in a sector of economic activity are distributed into occupations in the economy. Each occupation is associated with the knowledge items required for the performance of tasks. Consequently, the knowledge content of the economy is derived. As mentioned above, this is of particular interest given that the location of the knowledge content has only been attempted in terms of industrial organization (Andreeva & Kianto, 2011).

The Greek economy is chosen for the analysis due to its structural characteristics related to the weak domestic sectoral productive linkages (in manufacturing and high-tech industrial sectors), the relatively low international completeness, and the low level of industrial and technological development (Economakis et al., 2015). Furthermore, the Great Recession of 2008 was larger and deeper in Greece bequeathing high uncertainty and unemployment (Papapetrou & Tsalaporta, 2021; Petrakis, 2020). The analysis is concentrated in the year 2018 during which the Greek economy noted a stable improvement in economic performance (Bank of Greece, 2018). This selection was dictated mainly to avoid peculiarities related to the coronavirus disease 2019 (COVID-19 crisis) that characterized the following years, provided that the data used are collected with a 2-year lag.

The conclusions drawn from this study are relevant for the design of formal and informal educational policy to facilitate the adjustment process of the Greek production structure, especially in light of the fourth Industrial Revolution and the consequent technological change. Hence, the grounds for policymaking concern education, reskilling, and especially lifelong learning.

The remainder of this study is structured as follows; the “Human Capital and Economic Growth” section concerns a theoretical overview of human capital theory and its contribution to economic growth, while the “Toward a Better Understanding of Human Capital” section examines recent approaches to human capital. The “Technological Transformation, the Two Back-to-back Crises, and the Labor Market” section highlights the consequences of the economic crises on the labor market. The “Selected Evidence from the Greek Labor Market” section discusses the Greek production structure. The “Human Capital Analysis in Qualitative Characteristics” section describes the methodology for the evaluation of human capital based on its qualitative characteristics, while the “The Knowledge Content of the Greek Production Structure” section regarding the knowledge content of the Greek production structure for the year 2018. Finally, the “Conclusions and Further Research” section is dedicated to the conclusions and further implications for research.

### Human Capital and Economic Growth

The everlasting notion of human capital concerns the positive externalities for society and the economy arising from investing in people and improving their skills and education. Education and accumulation of human capital are acknowledged ever since classical thought as contributors to the enhancement of prosperity because they increase productivity and human capital quality (Miller, 1966).

For Smith (1937), work units were not only quantitative but also qualitative. At the microeconomic level, the division of labor results in specialization which leads to skills accumulation. Consequently, the acquisition of skills is being understood as a kind of “wealth” chained in the work units (Smith, 1937). On the same grounds, for Mill (1926) skills were economic utilities contributing to the accumulation of wealth but he did not consider them as “capital” since human beings do not consist a tradeable good. Hence, economic activity — through the accumulation of knowledge — is boosted by the dynamic returns of education as perceived by the competencies and qualities of all members of society. Later, Marshall (1948) defines personal wealth as those characteristics that make individuals industrially efficient.

The notion of “human capital” is attributed to Pigou (Blair, 2011) who mentioned it as a kind of investment. The theoretical approaches to human capital emerged in the 1950s and henceforth. Knight (1941) associates economic freedom with the accumulation of (human) capital, by improving which, the economy could overcome the diminishing returns of scale (Knight, 1944). Harrod (1943) links economic policymaking with human capital while Fisher (1946) emphasizes on the role of education as a tool of economic policy.

Chadwick highlights the importance of introducing systems of general education for the improvement of human capital (Ekelund & Price, 2012). He perceives human capital as an investment and that an effective and well-organized system of education, apart from increasing production, would reinforce the system of free competition against its internal disorganization (Miller, 1966). Correspondingly, Schultz (1961) and Mincer (1958, 1974) adopt a general concept of human capital where the role of education is decisive for improving productivity. The work of Becker (1964) organizes all the preexisting knowledge on human capital. Becker's two-period model addresses human capital accumulation as an investment decision that affects the individual’s future income (Becker, 1964, 1975). Whether an individual will choose to enhance their skills or not concerns a cost–benefit analysis regarding future earnings. Related models to Becker’s are those of Schultz (1961) and Mincer (1981). The common ground between the two approaches concerns the fact that the investment in human capital occurs on-the-job training, rather than through formal schooling (Blaug, 1976).

In an attempt for economic models to reflect the mechanism of complex economic phenomena such as economic growth, they endogenize human capital (Liening, 2013). This movement led to the emergence of endogenous growth theory. Endogenous growth models emerged during a time of technological changes that shifted the production needs of economies from needs for non-renewable resources to knowledge requirements (Laroche et al., 1999). Thus, the notion of human capital as reinforced through educational policy is linked to economic growth trying to explain the Solow (1956) residual.

The endogenous growth models are distinguished into two categories. In the first category, Lucas’s model (1988) is the most representative. In this model, the accumulation of human capital takes place either through education or through learning by doing. In this way, two different effects are exerted on economic development, an internal and an external productivity effect. For Lucas, generated knowledge is a mobilizing force for economic growth and it is an outcome of the investment in human capital.

In the second category, Romer’s model (1990) is the most representative. In this model, economic growth depends on the existing fund of human capital which, through investment in research and domain, generates new knowledge and facilitates the imitation of foreign technologies. Essentially, an economy’s starting point in terms of its level of knowledge is what determines the rate and extent of economic growth. In the same category of models belongs the study of Nelson and Phelps (1966) in which the capacity of economies to promote innovation is affected by human capital.

In essence, according to endogenous growth theory, economic growth is the outcome of endogenizing innovation, the intensity of which is positively correlated with the level of education, resulting in a significantly positive relation between education, innovation, and human capital (Aghion & Howitt, 1998).

### Toward a Better Understanding of Human Capital

Hitherto, the analysis concerned human capital as a fundamental factor of economic growth, especially through the prevailing generations of endogenous growth models. However, the endogenous growth models did not attempt to describe the features of human capital. Traditionally, the evaluation of human capital takes place based on quantitative characteristics (Laroche et al., 1999) referring to the years attended in school and lifelong education, and other indicators of investment in human capital (Hanushek et al., 2017).

Such approaches for the evaluation of human capital focus on the measurable quantitative impact of the investment in human capital in the improvement of productivity and life quality (Adelakun, 2011). On those grounds, Eicher’s model (1996) explains the relationship between human capital accumulation and technological change on the determination of relevant wages and economic development. Eicher (1996) concludes that the individual’s initial decision to invest in human capital finances the sector of education — raising demand for specialized labor — which in turn creates new technology and contributes to economic development.

Broadly, during the evaluation of human capital certain important issues crop up concerning the following facts (Laroche et al., 1999). First, human capital is non-tradable as it is embodied in individuals. Second, individuals do not always control the means and rate at which they generate human capital. Third, it may be general in kind, or specific. Fourth, it is broken into quantitative and qualitative features and fifth, human capital implicating knowledge, skills and technology, is subject to depreciation costs.

The evaluation of human capital through its qualitative characteristics is relatively recent and it is referring to the task approach to the labor markets (Acemoglu & Autor, 2011; Autor, 2013). The internal qualitative characteristics of human capital are (a) knowledge, (b) skills, (c) abilities, and (d) working activities. Tasks incorporated in the production process are related to the aforementioned qualitative characteristics of human capital, the labor market’s structure, and the prevailing technologies. In that respect, human capital refers to the accumulation of skills and productive knowledge which are incorporated in the productive factor of labor (OECD, 2001). That being said, the qualitative examination of human capital is cardinal when we evaluate skill-matching with the labor market requirements.

In this direction, business organization approaches perceive the firm as a complex economic institution emphasizing its internal resources rather than focusing only on the markets. Hence, the firm is not a “black box” as often has been stated by neoclassical economic theory. For example, in Penrose’s (1959) resource-based view, there are two types of knowledge contributing to the firm’s growth. These types of knowledge are (a) objective or transmissible knowledge which can be transmitted to all on equal terms and (b) expertise which is not transmitted (Penrose, 1959). Expertise depends on the historical path-dependence.

In terms of business organization, the firm determines the demand for labor according to three additional factors besides profit maximization. These factors are related with (a) the strategic planning of the firm; (b) the firm’s demand for skills, knowledge, expertise, etc.; and (c) the internal processes for the determination of the wages (Fernández-Huerga, 2019). Thus, businesses are shaping the labor demand and affect the accumulation of human capital directly (on the job training) and indirectly (by affecting worker’s expectations).

Human capital is a dynamic notion that depends on the characteristics of the environment. For example, innovative activity generates competitive advantages for the firm (Schumpeter, 1934) while the effects of networking are equally important for the quantity and quality of knowledge accumulated (Farace & Mazzotta., 2015) since workers interact with each other within and across firms (Eppelsheimer et al., 2022). That being said, human capital does not only concern us about the individual but also in relation to the social conditions in terms of which it is occasioned and accumulated.

Spatial analysis on human capital has indicated that distance may negatively affect the human capital externalities due to (a) increased cost of planned interactions and (b) reduced likelihood of unintended encounters leading to the exchange of knowledge (Eppelsheimer et al., 2022). It is found that higher ability individuals benefit more from these encounters and thus they locate in larger cities which exhibited better idea-exchange opportunities (Davis & Dingel, 2019). Besides distance, the general social and cultural framework constitutes important factors for knowledge spill-overs.

In this direction, the concepts investigated are social capital (Coleman, 1988) and cultural capital (Bourdieu, 1986) and how they operate and interact with human capital. Social capital refers to the interpersonal relations formed within society (Coleman, 1988). Thus, the analysis includes concepts such as social norms, institutions, social networks, and the importance of information. Cultural capital refers to the cultural institutions transferred from generation to generation (Schuller, 2001). Ultimately, human capital is a social activity and emerges out of the interaction of ideas and individuals (Laroche et al., 1999).

It is ascertained that human capital and its accumulation could impact the rate of growth of an economy. For instance, the quality of human capital, as well as the rate of intensity of technological change, may explain the non-convergence of incomes from one country to another (Easterly & Levine, 2001; Hall & Jones, 1997). The intensity and adaptivity to developments of the qualitative characteristics serve economic growth (Nelson & Phelps, 1966; Romer, 1986, 1990). Therefore, labor markets around the world are called to adjust to the endogenous and exogenous developments with the adjustment process varying subsequently due to their structural characteristics. The following section discusses the consequences of the two major economic crises (Great Recession of 2008 and the COVID-19 pandemic) to the labor market in Greece and around the globe.

### Technological Transformation, the Two Back-to-back Crises, and the Labor Market

Economic crises disrupt the labor market’s organization and efficiency by altering the production structure’s characteristics leading to shifts in the relative demand for occupations and, consequently, human capital. The consequences of a crisis in the labor market can be observed through the tables of labor demand worldwide due to the reshuffles and shifts of the relative importance of the occupations within the economy. Broadly, a serious disturbance bequeaths high uncertainty (Bloom, 2009) impacting all aspects of economic life and the investment decisions of individuals (Bloom, 2009; Knight, 1921). The impact of an economic crisis differs from country to country as the institutional framework, stabilizing mechanisms, and fiscal policy vary substantially (Daly et al., 2014).

One of the consequences of economic crises is unemployment which varies depending on the circumstances. Circular unemployment emerges from the decline in economic activity, while structural unemployment refers to the skill-mismatch in the labor market. Structural unemployment arises because some occupations are experiencing a boost while others experience a decline. Such developments accord with the prevailing trends in the labor market locally and around the globe.

Nowadays, the labor market in Greece and worldwide is affected by the fourth Industrial Revolution and the COVID-19 crisis (Petrakis, 2020). Thus, the long-term forces of the fourth Industrial Revolution and the short-term consequences of the shock by COVID-19 are interwoven. The impact of the technological change was distinct since 2010 combined with the effects of globalization. The Great Recession of 2008 emerged in tandem with the effects of technological change and highlighted the need for adjustment to a more technologically advanced economy.

The fourth Industrial Revolution and the technological change impact all the sectors of economic activity changing the nature of tasks and therefore occupations (Schwab, 2017). On the supply side, the introduction of new technologies generates new production processes and competition circumstances as well. As a result, firms are called to quickly and efficiently adapt to the developments occurring in the labor market. On the demand side, the shifting of consumption preferences further pressures the firms to adjust their goods and services.

Meanwhile, the COVID-19 crisis burst out in waves resembling “entangled webs” (Baldwin et al., 2020) causing a sudden stop to the global economy only comparable with the Great Depression of 1929 and the Great Recession of 2008 (Petrakis, 2020). Regarding the labor market, the COVID-19 crisis highlighted the importance of the adjustment to the demands of the fourth Industrial Revolution, because digital economy and working-from-a-distance were included in the government response for the mitigation of the crisis (Huang et al., 2021; Petrakis, 2020).^2^ Yet, many occupations were affected by work-from-a-distance and the overall reduction in job availability or the automation of labor (Petrakis, 2020).

The effect of technological change on the production factor of labor occurs through (a) labor augmenting technological advances; (b) automatization; (c) deepening of automatization; and (d) creation of new tasks (Acemoglu & Restrepo, 2018). Automatization creates a productivity effect and a displacement effect (Acemoglu & Restrepo, 2018). Which of the two will prevail depends on the production structure and the followed policies (Bessen, 2019). Hence, a smooth transition in the labor market lies in the policy responses because automation both creates and replaces jobs. The risk for employment, especially, lies in low-intensity automation that displaces low-skill jobs. For this reason, Acemoglu and Restrepo (2018) argue that the most effective measure for the displacement effect is to generate new labor-intensive tasks to combat structural unemployment.

The risk of automation of a job concerns whether technology-intensive capital could replace labor-intensive capital in both routine tasks and non-routine tasks (Blien et al., 2021; Frey & Osborne, 2017). The replacement of routine tasks by automation is connected with the “polarization phenomenon” (Autor et al., 2006) in which, middle-skilled jobs are displaced and labor demand concentration occurs in the poles, namely low-skilled and high-skilled jobs.

The intensity of the tasks and whether they concern (a) reception and manipulation; (b) creative intelligence; and (c) social intelligence dictate the realization of the risk of automation (Frey & Osborne, 2017). Hence, jobs characterized by tasks that require critical thought, social skills, and creativity are of low-replacement risk by technology-intensive capital (Autor, 2015). This observation reserves an important role for creativity-and-digitally oriented economies where profit maximization depends on originality and the institutional framework for copyright protection.

In essence, technological change and the fourth Industrial Revolution generate new tasks and jobs and consequently fermentations causing rearrangements in the labor market which intensify inequalities and skill-mismatch (Tyson, 2021). The absence of jobs for all the skills levels results in job scarcity because middle-skilled workers replace low-skilled workers crowding them out of the labor market (Tyson, 2021). Hence, technological change could crow-out workers and suppress the middle class (Moro et al., 2021).

Except for technological change, another important issue in the labor market is the promotion of sustainability for the sustainable integration of the firms and the attainment of sustainable development goals. In this direction, covering the skill-mismatch, correspondingly to the sustainable development goals, is the most important step for a transition toward a green economy (European Centre for the Development of Vocational Training, Cedefop, 2010). Green economy generates jobs for all skill levels at a fast pace leading to the improvement of skills and education (European Commission, 2019). In general, the reduction of inequalities, the insurance of human rights, the supply of long-term positions, the combat of structural unemployment due to skill-mismatch, and the promotion of green economy are at the center of the sustainable integration in the labor market (Jianu et al., 2021).

Thus, re-skilling and up-skilling are crucial for the resilience (Holling, 1973) of the labor market. Resilience refers to the adjustability of the labor market to the economic shocks (Chapple & Lester, 2010; OECD, 2012). In this direction, Moro et al. (2021) based on data from the O*NET database (which is also used in this study) create a model which locates occupational mobility according to skill requirements similarity. Labor markets with overlapping skill requirements generate positive endogenous effects empowering the resilience of the labor market (Moro et al., 2021). If an occupation is negatively affected, other occupations with similar skill requirements absolve the displaced workers with low-cost retraining. Hence, the density of occupational interconnections promotes labor market resilience and higher real wages (Moro et al., 2021).

The above discussion illustrates that the labor market concerns of a range of dimensions related to the structure of human capital and the density of occupational interconnections. It is evident that the study of the labor market is crucial in order to extract conclusions regarding (a) the transformation and skills requirements, (b) the effects of technological transformation on the nature of tasks, and (c) the effective utilization of funds for the training and education of the workforce. From this point on, the implications of policymaking are complex. Particular emphasis should be placed on educational policy conclusions since they identify opportunities for targeted interventions in the education system to provide the knowledge necessary for the development of advanced production systems.

### Selected Evidence from the Greek Labor Market

Worldwide and especially in Greece, the Great Recession of 2008 brought an increase in circular and structural unemployment (Guichard & Rusticelli, 2010; Papapetrou & Tsalaporta, 2021). For Greece, the year 2011 — one of the most severe years of the economic crisis — was characterized by high unemployment, especially among youth (40.8%) while 7 years later, in 2017, a significant part of the population (15.3%) was long-term unemployed (European Commission, 2018a). In general, the effects of the economic crisis were unevenly distributed in the local labor market, due to both exogenous and structural factors (Palaskas et al., 2015). These conditions dictated the structural reforms that occurred from 2009 to 2016 (Bank of Greece, 2018; European Commission, 2014).

Structural reforms concerned the improvement of pathogenic causes of the Greek crisis. Hence, they were mainly related to measures for the improvement of the effectiveness of public administration, judicial system, the use of land in general, and the collection of taxes (Bank of Greece, 2018). Furthermore, in light of the fourth Industrial Revolution, structural reforms of this period aimed at the technological and digital integration of the economy. Hence, the adjustment to technological developments resulted in an increased demand for skills related to high digital literacy (Popović et al., 2016). The demand for these skills was different among different occupations and sectors of economic activity, demographic groups, and geographic areas (Finneran, 2015). Nonetheless, the most important drivers of change in the labor market fall into two categories: demographic and socioeconomic, and technological (Popović et al., 2016). Demographic factors concern changes in the nature of the occupations, the role of the middle class, climate conditions, geopolitical situations, and population aging (Popović et al., 2016). They exert pressure on the labor market and also receive pressure with observable repercussions in macroeconomic terms. Technological factors refer to the introduction of new technologies, data management and processing technologies, robotics, and artificial intelligence (Popović et al., 2016).

Occupational shifts from 2011 to 2019 for Greece are presented in the following Table 1.

**Table 1:** Occupations with shifts in employment in the Greek economy (2011–2019)

Occupation	Difference 2011–2019 (thousands)
Technicians in building and construction industry, except electricians	− 79,770
Specialist farmers and stockbreeders	− 58,047
Cleaners and helpers	− 39,429
Managers of hotels, restaurants, retail and wholesale trading, and other businesses	− 38,753
Other office staff	− 35,702
Technicians in the positive sciences and engineering	− 25,247
Technicians in metals, machinery, and similar occupations	− 23,949
Salespeople	− 23,347
Technicians in food processing, wood processing, the processing of clothing items, and related occupations	− 19,831
Professionals in information and communications technology	9871
Customer service personnel	10,082
Professionals in individual personal care	10,764
Employed in security services	10,869
Drivers of transport vehicles and handlers of mobile equipment	16,342
Assistants to business and management professionals	16,766
Professional workers in the health sector	24,106
General duty clerks and keyboardists	53,031
Employed in the provision of personal services	54,101

The data in Table 1 originate from the Work Force study of the Hellenic Statistical Authority (unpublished). The shifts in occupations indicate a general turn toward the digital and service economy. More specifically, the occupations favored related to service provision (personal care, clerics and keyboardists, health professionals, professionals in businesses and management, drivers, customer service personnel, and professionals in information and communications technology). On the other hand, the occupations that experienced the greatest decline in their activity concerned technicians in the construction industry, professional farmers, professionals working in the service provision (cleaners and helpers; managers of hotels, restaurants, and other services; non-specialized office staff; salespeople), and technicians.

These shifts in occupations concern the negative implications of the Great Recession of 2008. An interesting point to note is that even with the strike of the COVID-19 crisis, the Greek economy little was oriented toward the digital economy. Developmental prospects of the Greek economy for 2019 were especially highlighted the need for adaptation to the digital economy (Bank of Greece, 2018). This fact lines up with the forecasts that indicate an increased demand for occupations related to artificial intelligence, machine learning, data specialists, and digital marketing and strategy specialists (Alekseeva et al., 2021).

The outline of the Greek production structure indicates an economy with low- and middle-specialization products (Petrakis, 2020). The Greek economy is service-oriented (including the public sector) while manufacturing activity tends to feature lower total multipliers (Backinezos et al., 2020). Specifically, according to Danchev et al. (2014), trade and tourism activities contribute to 23.3% of overall value-added in the economy along with public administration and education sectors. The sectors of agriculture, forestry, and fishing contribute less than 3.5% in gross value added yet employ 13% of the workforce while similar observations apply to the manufacturing sector (Danchev et al., 2014).

Another important aspect of the labor market is the labor dynamism of the sectors of economic activity which is expressed not only by the contribution of the sectors to production but also by the contribution of the sectors to employment (Kaminioti, 2014), and by the quality of interconnections within the labor market (Moro et al., 2021). In this respect, the Greek labor market lacks dynamism (Bulman, 2020) and competitiveness as a result of rather structural than cost factors (Economakis et al., 2015). Structural factors refer to the lack of connections between the sectors of high productivity and those sectors that are technologically backward (Mouzelis, 1978). Besides, according to the digital economy and society index (European Commission, 2018b), Greece ranked penultimate among the 28 European Union member States. Furthermore, Greece is of low economic complexity as it exports goods that in their majority are of little diversity and not sophisticated.

Let it be noted that these characteristics of the Greek production structure are not unique. Countries such as Portugal, Spain, and Italy present similar characteristics as they are defined by a certain socioeconomic model (Andreotti et al., 2001; Karamessini, 2008). Economic development in these countries is based on small businesses and the big public sector (Kádár, 1975). Furthermore, these countries present low and middle specialization and skills in the workforce (Karamessini, 2008). That being said, the assessment of the Greek production structure in terms of its knowledge content is expected to present similarities with the aforementioned countries. However, this is ground for further research and probable comparisons between the European South and North. If knowledge content differences exist between the two production structures, such findings could explain their divergence in terms of productivity and economic complexity.

Having highlighted the outline of the Greek production structure, it is of interest to examine the knowledge content within the economy. By the term *knowledge content*, the present study refers to the concentration of the knowledge items required to carry through a series of occupations. The definition of each knowledge item — originating from the O*NET database — is given in Table A1 of the Appendix. The following section is dedicated to the methodology for the determination of the knowledge content within the Greek economy.

### Human Capital Analysis in Qualitative Characteristics

Following the prevalence of human capital as a determining factor of economic growth, economists assess human capital through the measurement of its externalities. Hence, human capital is measured by means of globally statistical variables such as the impact of schooling on productivity and economic growth (Lucas, 1988), and the positive impact on health, fertility, and the reduction of child mortality (Lewin et al., 1983; Woodhall, 2001). However, there is criticism regarding the effectiveness of these indicators for identifying the relationship between education and economic growth (Petrakis & Stamatakis, 2002).

By contrast to these approaches, the present study analyzes human capital through its qualitative characteristics (knowledge, skills, abilities, and working activities) focusing on the knowledge feature of human capital. The analysis is concentrated on the year 2018 since detailed data regarding the long-term consequences of the COVID-19 crisis on the labor market are yet unavailable. The data (unpublished) originate from the Workforce Study of the Hellenic Statistical Authority.

The point of departure for the proposed methodology is the relationship between employment and occupations operating in the (Greek) economy. Table 2 distributes the employment among the various sectors of the economy.

**Table 2:** Employment in the sectors of economic activity in Greece (2018)

Sectors of economic activity	Employees
Wholesale and Retail Trading, Vehicle and Motorcycle Repairs	690,093
Farming, Forestry and Fishing	472,530
Provision of Food and Lodging	382,921
Processing	359,718
Public Administration and Defense, Mandatory Social Security	330,255
Education	307,668
Health and Social Welfare Services	244,763
Professional, Scientific and Technical Activities	217,019
Transport and Storage	183,422
Constructions	151,306
Information and Communication	98,194
Administrative and Support Services	88,861
Fiscal and Insurance Activities	86,228
Other Activities in Service Provision	82,189
Arts, Culture and Entertainment	52,.122
Water Provision, Waste Management, and Sanitation	32,178
Provision of Energy and Air-conditioning	32,125
Activities of Households as Employers	30,776
Mines and Quarries	11,176
Real Estate Management	4939
Activities of Overseas Organizations and Agencies	1912
Total	3,860,395

 Afterwards, each occupation in the economy is matched with the sector within it operates to create a table that describes the relationship between occupations and sectors of economic activity (Table A2 of the Appendix).^3^ In the sequel, the knowledge items from the O*NET database are being matched with the occupations in the economy. The O*NET database matches the knowledge items with the occupations according to a ranking of importance.^4^ The knowledge items do not indicate the level of specialization but the presence of various levels of knowledge, depending on the occupation.

In the sequel, each occupation of Table A2 is matched with the three most important knowledge items. Furthermore, depending on the sector within an occupation operates, there is an extra (sector) specific knowledge item which is determined by subjective criteria based on the structural characteristics and the nature of the sector. The sector-specific knowledge item is particularly useful when two occupations share the same knowledge items but belong to different sectors. In this case, the sector-specific knowledge item differentiates the knowledge concentration. Eventually, all occupations are being matched with four knowledge items (three basic and one sector-specific). Initially, a routine was developed in Visual Basic to match the occupations and sectors with the sector-specific knowledge item. In the sequence, the data were processed by means of Excel using pivot table sorting resulting in the final aggregate table of the knowledge content.

**Fig. 1:**
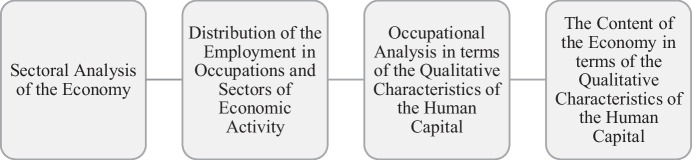
Human capital analysis in qualitative characteristics

Thus to investigate the knowledge content of the production structure, the methodology followed comprises the sectoral analysis along with a synthesis of the knowledge requirements for every occupation employed in the economy. The identification of the knowledge content density and its evaluation is difficult to achieve (Abreu et al., 2010), while it has been only attempted in terms of industrial organization (Andreeva & Kianto, 2011). Consequently, this study contributes in literature by proposing an alternative way for the description and evaluation of the knowledge content within the economy. Figure 1 below describes the steps of the methodology.

### The Knowledge Content of the Greek Production Structure

As mentioned above the point of departure for the analysis is the relationship between employment and the sectors of economic activity which is presented in Table 2 above. Broadly, the method used is summarized in the following Figure 1 which describes the steps followed.

For Greece for the year 2018, the sectors with the greatest employment concentration were those of (a) *Wholesale and Retail Trading, Vehicle and Motorcycle Repairs*; (b) *Farming, Forestry and Fishing*; (c) *Provision of Food and Lodging*; (d) *Processing*; (e) *Public Administration and Defense, Mandatory Social Security*; (f) *Education*; (g) *Health and Social Welfare Services*; and (h) *Professional, Scientific and Technical Activities*. These findings correspond with the outline of the Greek production structure which was discussed in the abovementioned section.

An interesting exercise on the use of the O*NET knowledge items would be to generally match these sectors of economic activity with their corresponding knowledge characteristics. Under this notion, one can refer to knowledge requirements as well.

Therefore, an example reference could be made to the sectors of *Wholesale and Retail Trading and Vehicle Repairs*, and the sector of *Provision of Food and Lodging* where similar knowledge items are required but there are also differences related to the tasks undertaken. For example, for both sectors, important knowledge items could be (a) practical application of *mathematics* for carrying out everyday transactions with customers, (b) *customer and personal service* to ensure quality service to clients, (c) *sales and marketing* knowledge for the effectiveness of the sales practices, (d) *clerical knowledge* and (e) *administration and management* for drawing sales plan and managing a budget, and (f) *English language* in the context of communicating with foreign customers. Certainly, in the sector of *Provision of Food and Lodging*, different emphasis is placed in the specialization on some of the abovementioned knowledge characteristics, while there is no required knowledge on machine operation and repair or engineering in general.

On the other hand, the sector of *Farming, Forestry and Fishing* requires knowledge of applied *biology* for the appropriate care of nature and animals, as well as knowledge in *engineering and technology* for the correct use and maintenance of the farming equipment. The sector of *Processing* requires technical knowledge related to *mechanical, engineering and technology* knowledge items for the operation of machinery.

For the sector of *Public Administration and Defense and Mandatory Social Security*, important knowledge items for better carrying out its tasks could be (a) *clerical knowledge*; (b) *administration and management*; (c) *English language*; (d) *economics and accounting*; and (e) knowledge on *law, government and jurisprudence*, as well as (f) *education and training* knowledge to educate the workforce employed.

Relevant knowledge items for the sector of *Education* could be (a) *education and training*, (b) *mathematics*, and (c) *English language* and generally knowledge items that require specialized knowledge to be taught according to the curricula such as (d) *chemistry*, (e) *biology*, and (f) *foreign language*. The knowledge items for the sector of *Education* could be of similar importance to those in the sector of *Professional, Scientific and Technical Activities* differentiated only by the tasks of the latter sector. Finally, for the sector related to *Activities in Human Health and Social Welfare*, important knowledge items could be (a) *biology*, (b) *chemistry*, (c) *medicine and dentistry*, and (d) *English language*.

From the aforementioned examples, it is understood that a plethora of occupations and consequently sectors use similar knowledge items but in different intensity and specialization. This is, of course, a rational and expected assumption which is related to two issues.

**Table 3:** Knowledge requirements of the Greek production structure (2018)

Knowledge item	Employees	Percentage
Mathematics	1,541,004	10.74%
Customer and personal service	1,342,052	9.35%
Clerical	1,148,886	8.01%
Mechanical	1,082,050	7.54%
Sales and marketing	1,040,750	7.25%
English language	709,687	4.95%
Biology	709,717	4.95%
Administration and management	664,260	4.63%
Engineering and technology	656,154	4.57%
Education and training	656,154	3.95%
Chemistry	529,495	3.69%
Food production	476,755	3.32%
Production and processing	439,980	3.07%
Transportation	426,945	2.98%
Economics and accounting	378,473	2.64%
Computers and electronics	351,856	2.45%
Public safety and security	332,248	2.32%
Geography	286,532	2.00%
Medicine and dentistry	236,618	1.65%
Personnel and human resources	234,643	1.64%
Therapy and counseling	206,610	1.44%
Design	187,403	1.31%
Law, government, and jurisprudence	156,325	1.09%
Building and construction	154,721	1.08%
Phycology	119,961	0.84%
Telecommunications	91,262	0.64%
Communications and media	62,588	0.44%
History and archeology	62,323	0.43%
Physics	45,900	0.32%
Sociology and anthropology	39,843	0.28%
Fine arts	34,940	0.24%
Foreign language	33,086	0.23%

 Firstly, the ability to activate the productivity via education and re-skilling to support the occupations within the sectors. Secondly, the assessment of the interconnections within the labor market and the sectors’ dynamism.^5^

The knowledge content in the Greek production structure is traced using the methodology described in the “Human Capital Analysis in Qualitative Characteristics” section. Thus, Table 3 presents each knowledge item and the number of the employees (absolute numbers and percentages) who use these knowledge items to effectively put through the tasks related to their occupations. The knowledge items in Table 3 are ranked by importance according to their employment density.

The top 10 knowledge items for the Greek production structure for the year 2018 are (a) *mathematics*, (b) *customer and personal service*, (c) *clerical*, (d) *mechanical*, (e) *sales and marketing*, (f) *English language*, (g) *biology*, (h) *administration and management*, (i) *engineering and technology*, and (j) *education and training*. Hence, these ten knowledge items that approximately correspond to 66% concern a simple rather than sophisticated knowledge background, a finding reflected in the Greek production structure specialization which is of low and middle levels (Petrakis, 2020).

## Conclusions and Further Research

This study proposes an alternative way for the evaluation of human capital based on its qualitative characteristics rather than the measurement of its positive externalities. Following the division of human capital into (a) knowledge, (b) skills, (c) abilities, and (d) working activities, it focuses on the first component in order to illustrate the knowledge content of the Greek production structure.

To that end, the employment concentration of the Greek production structure is matched with the knowledge items from the O*NET database according to the described methodology. The analysis of the knowledge content is bounded to four knowledge items (three basic and one sector-specific). Nevertheless, the knowledge items used per occupation are greater than these four; however, if more than four knowledge items were included, the analysis would be of high complexity and the subjective criteria of the sector-specific knowledge item might cause uncertainty in the interpretation of the results.

For Greece for the year 2018, it is found that the ten most important knowledge items which correspond to 66% of the total are (a) *mathematics*, (b) *customer and personal service*, (c) *clerical*, (d) *mechanical*, (e) *sales and marketing*, (f) *English language*, (g) *biology*, (h) *administration and management*, (i) *engineering and technology*, and (j) *education and training*. It is, hence, ascertained that given the structural characteristics of the Greek production structure, the basic knowledge background of the Greek economy does not include sophisticated knowledge requirements in their majority.

The proposed assessment of the knowledge content of the production structure brings into light certain conclusions. First, that the analysis of human capital is not a merely theoretical process but produces a quantified description of its internal characteristics in the total of the economy, something which was only attempted in terms of the firm (Andreeva & Kianto, 2011). Second, that each knowledge item, depending on the occupation and sector of economic activity, differs in terms of specialization and relative importance. Third, the assessment of the knowledge content of a production structure refers to employed individuals who have already completed their formal education, or to individuals who wish to reenter the labor market (if we are referring to the unemployed). Therefore, such an analysis of human capital produces robust and significant information regarding the labor market and the areas of policy intervention for the improvement of human capital and boost of employment in sectors of high importance.

In that respect, the issue of the diversification of the production process and the improvement of productivity in Greece, concerns the advancement of the qualitative characteristics of the human capital through the educational system (Bulman, 2020). Nevertheless, productivity improvement is greater when the economy is technologically progressive (Nelson & Phelps, 1966). Hence, the adaptation to new technologies and the re-skilling and up-skilling of the workforce is a vital parameter for the Greek economy.

Let it be noted that a non-sophisticated knowledge content (as found in the present study) is accompanied by more favorable conditions for an upgrade rather than a sophisticated one. The upgrade of a sophisticated knowledge content would require depreciation of a high level of knowledge — the case of the Digitalization of the Baltics (International Economic Forum, 2019) is illuminating on this point. Provided that in the Greek economy, the existing knowledge is of a middle and low level, the improvement in productivity of the existing production structure will not involve a particularly high cost.

Pointedly, the role of education is crucial, as it contributes decisively to the smooth transformation of a production structure given that the knowledge accumulation and skills formation take place under formal and lifelong learning (Brunello & Wruuck, 2019; European Commission, 2018c). Furthermore, the “demand for knowledge” is correlated with the “availability of knowledge” (Kalantzis & Cope, 2013) and as a result education. Thereafter, the analysis concerns the possibility of intervention in those specific knowledge areas which could serve to improve the productivity of the existing production structure.

That being said, the grounds for policymaking concern education, reskilling, and especially lifelong learning. Considering the difficulty to support formal education through the raising of public expenditure as well as the constant technological advancements, respected and certified lifelong learning programs are crucial because they incorporate both past (necessary) and new knowledge according to the job requirements and descriptions (Bulman, 2020; Giouli et al., 2021; Tyson, 2021). Furthermore, this kind of educational structure adjusts accordingly to changes in the labor market almost immediately and effectively due to their main characteristics of organizational flexibility and low operational cost (Giouli et al., 2021).

A more radical transformation of the productive structure requires a higher order of analysis because the alteration of the production model, by introducing higher knowledge requirements, requires time, and educational and training investments of a bigger scale. Such intervention would be the re-organization of formal education curricula and the promotion of preferences toward occupations in both general and vocational^6^ tracks to ensure the labor market’s resilience. Nevertheless, the analysis offered here may assist in locating such investments.

This study delimits further research in two interesting directions. First, the analysis of the Greek production structure to the remainder of the qualitative characteristics of human capital. Second, the analysis of human capital in terms of qualitative characteristics in countries of the European South and North to define whether the differences in productivity and economic complexity correspond to differences in the qualitative characteristics of the human capital.

## Supplementary Information

Below is the link to the electronic supplementary material.

Supplementary file1 (DOCX 83 KB)

## Footnotes

1 The O*NET database is based in the USA providing information on the knowledge, skills, abilities, and working activities required to perform a range of key occupations. Furthermore, it contains information on the demand for the quality characteristics of human capital in the economy, the average salary earnings per profession, and automated questionnaires for vocational and professional guidance. The database is accessible through this link (https://www.onetcenter.org/database.html). In this study, O*NET is used as a reference for the description of the knowledge characteristics.

2 Let it be noted that the long-term consequences on the quality of human capital from the closing down of educational institutions have not been assessed at the time being (Wren-Lewis, 2020).

3 Some occupations operate in more than one sector; hence, the employment concentration of the corresponding occupations to the sector is taken into account in order to leave intact the total number of employees distributed across the occupations and sectors.

4 For example, according to O*NET (https://www.onetonline.org/link/summary/11-2021.00) for the occupation of *Marketing Managers*, the knowledge items required ranked by importance are *sales and marketing*, *English language*, *administration and management*, *customer and personal service*, *communications and media*, *computers and electronics*, *mathematics*, *design*, *economics and accounting*, and *education and training*.

5 This is an interesting finding and of immense importance regarding the Greek economy for the support of some sectors given that the negative forecasts for 2030 indicate that the sectors with the greatest decline in their economic activity would be the primary and secondary sectors of economic activity related to agriculture, fishing, and manufacturing (European Centre for the Development of Vocational Training, Cedefop, 2020).

6 According to Silliman and Virtanen (2022) for Finland, admission to the vocational track increases annual income by 7% at age 31, and the benefits show no signs of diminishing with time. Furthermore, they find that admission to the vocational track does not increase the likelihood of working in jobs at risk of replacement by automation or offshoring.
