# Supplementary material for: The Knowledge Content of the Greek Production Structure in the Aftermath of the Greek Crisis
Source: J Knowl Econ. 2023 Jan 31:1–22. Online ahead of print. doi: 10.1007/s13132-022-01095-7 (PMC9887571; doi:10.1007/s13132-022-01095-7)
Supplement: Supplementary file 1 — Supplementary file1 (DOCX 83 KB) [file 13132_2022_1095_MOESM1_ESM.docx]

**Appendix**

**Table A1**. Description of the Knowledge Items from the O*NET Database

| Knowledge Item | Description |
| --- | --- |
| Administration and Management | Knowledge of principles and processes involved in business and organizational planning, coordination, and execution. This includes strategic planning, resource allocation, manpower modeling, leadership techniques and production methods |
| Biology | Knowledge of plant and animal organisms, their tissues, cells, functions, interdependencies, and interactions with each other and the environment. |
| Building and Construction | Knowledge of materials, methods, and the tools involved in the construction or repair of houses, buildings, or other structures such as highways and roads. |
| Chemistry | Knowledge of the chemical composition, structure, and properties of substances and of the chemical processes and transformations that they undergo. This includes uses of chemicals and their interactions, danger signs, production techniques, and disposal methods. |
| Clerical | Knowledge of administrative and clerical procedures and systems such as word processing, managing files and records, stenography and transcription, designing forms, and other office procedures and terminology. |
| Communications and Media | Knowledge of media production, communication, and dissemination techniques and methods. This includes alternative ways to inform and entertain via written, oral, and visual media. |
| Computers and Electronics | Knowledge of circuit boards, processors, chips, electronic equipment, and computer hardware and software, including applications and programming. |
| Customer and Personal Service | Knowledge of principles and processes for providing customer and personal services. This includes customer needs assessment, meeting quality standards for services, and evaluation of customer satisfaction. |
| Design | Knowledge of design techniques, tools, and principles involved in production of precision technical plans, blueprints, drawings, and models |
| Economics and Accounting | Knowledge of economic and accounting principles and practices, the financial markets, banking and the analysis and reporting of financial data. |
| Education and Training | Knowledge of principles and methods for curriculum and training design, teaching and instruction for individuals and groups, and the measurement of training effects. |
| Engineering and Technology | Knowledge of the practical application of engineering science and technology. This includes applying principles, techniques, procedures, and equipment to the design and production of various goods and services. |
| English Language | Knowledge of the structure and content of the English language including the meaning and spelling of words, rules of composition, and grammar. |
| Fine Arts | Knowledge of the theory and techniques required to compose, produce, and perform works of music, dance, visual arts, drama, and sculpture. |
| Food Production | Knowledge of techniques and equipment for planting, growing, and harvesting food products (both plant and animal) for consumption, including storage/handling techniques. |
| Foreign Language | Knowledge of the structure and content of a foreign (non-English) language including the meaning and spelling of words, rules of composition and grammar, and pronunciation. |
| Geography | Knowledge of principles and methods for describing the features of land, sea, and air masses, including their physical characteristics, locations, interrelationships, and distribution of plant, animal, and human life. |
| History and Archaeology | Knowledge of historical events and their causes, indicators, and effects on civilizations and cultures. |
| Law, Government and Jurisprudence | Knowledge of laws, legal codes, court procedures, precedents, government regulations, executive orders, agency rules, and the democratic political process. |
| Mathematics | Knowledge of numbers, their operations, and interrelationships including arithmetic, algebra, geometry, calculus, statistic and their applications |
| Mechanical | Knowledge of machines and tools, including their designs, uses, repair, and maintenance. |
| Medicine and Dentistry | Knowledge of the information and techniques needed to diagnose and treat human injuries, diseases, and deformities. This includes symptoms, treatment alternatives, drug properties and interactions, and preventive health-care measures. |
| Personnel and Human Resources | Knowledge of principles and procedures for personnel recruitment, selection, training, compensation and benefits, labor relations and negotiation, and personnel information systems. |
| Philosophy and Theology | Knowledge of different philosophical systems and religions. This includes their basic principles, values, ethics, ways of thinking, customs, practices, and their impact on human culture. |
| Physics | Knowledge and prediction of physical principles, laws, their interrelationships, and applications to understanding fluid, material, and atmospheric dynamics, and mechanical, electrical, atomic and sub- atomic structures and processes. |
| Production and Processing | Knowledge of raw materials, production processes, quality control, costs, and other techniques for maximizing the effective manufacture and distribution of goods. |
| Psychology | Knowledge of human behavior and performance; individual differences in ability, personality, and interests; learning and motivation; psychological research methods; and the assessment and treatment of behavioral and affective disorders. |
| Public Safety and Security | Knowledge of relevant equipment, policies, procedures, and strategies to promote effective local, state, or national security operations for the protection of people, data, property, and institutions. |
| Sales and Marketing | Knowledge of principles and methods for showing, promoting, and selling products or services. This includes marketing strategy and tactics, product demonstration, sales techniques, and sales control systems |
| Sociology and Anthropology | Knowledge of group behavior and dynamics, societal trends and influences, human migrations, ethnicity, cultures and their history and origins. |
| Telecommunications | Knowledge of transmission, broadcasting, switching, control, and operation of telecommunications systems. |
| Therapy and Counseling | Knowledge of principles, methods, and procedures for diagnosis, treatment, and rehabilitation of physical and mental dysfunctions, and for career counseling and guidance. |
| Transportation | Knowledge of principles and methods for moving people or goods by air, rail, sea, or road, including the relative costs and benefits. |

Table A2 analyzes the relationship between employment and professions for 2018 and emerges from data derived from the Hellenic Statistical Authority (Efstratoglou Labor INE/GSSΕ). It presents data for a two-digit analysis of sectors in the down columns and a three-digit analysis of professions across. Out of this table emerge the factors of the correspondence between the number of employees in each profession who are employed in each sector and the reverse.

**Table A2:** Occupations in the Sectors of the Greek Economy (2018)

| 1. Employed by three-digit profession and one-digit sector of economic activity | Single-digit categories of economic activity | | | | | | | | | | | | | | | | | | | | | |
| --- | --- | --- | --- | --- | --- | --- | --- | --- | --- | --- | --- | --- | --- | --- | --- | --- | --- | --- | --- | --- | --- | --- |
|  | Total | Farming, Forestry and Fishing | Mines and Quarries | Processing | Provision of Energy and Air-conditioning | Water Provision, Waste Management and Sanitation | Constructions | Wholesale and Retail Trading, Vehicle and Motorcycle Repairs | Transport and Storage | Provision of Food and Lodging | Information and Communication | Fiscal and Insurance Activities | Real Estate Management | Professional, Scientific and Technical Activities | Administrative and Support Services | Public Administration and Defense, Mandatory Social Security | **Education** | Health and Social Welfare Services | Arts, Culture and Entertainment | Other Activities in Service Provision | Activities of Households as Employers | Activities of Overseas Organizations and Agencies |
|  | Count | Count | Count | Count | Count | Count | Count | Count | Count | Count | Count | Count | Count | Count | Count | Count | **Count** | Count | Count | Count | Count | Count |
| Total | 3860395 | 472530 | 11176 | 359718 | 32125 | 32178 | 151306 | 690093 | 183422 | 382921 | 98194 | 86228 | 4939 | 217019 | 88861 | 330255 | **307668** | 244763 | 52122 | 82189 | 30776 | 1912 |
| 111 Members of legislative bodies and senior administrative members | 964 | 0 | 0 | 0 | 0 | 0 | 0 | 0 | 0 | 0 | 0 | 0 | 0 | 0 | 0 | 964 | **0** | 0 | 0 | 0 | 0 | 0 |
| 112 Chief executive officers and general managers | 2059 | 0 | 0 | 479 | 0 | 0 | 0 | 1074 | 175 | 0 | 0 | 0 | 0 | 0 | 178 | 0 | **153** | 0 | 0 | 0 | 0 | 0 |
| 121 Business service managers and administrative managers | 7568 | 0 | 0 | 2660 | 0 | 138 | 0 | 335 | 0 | 254 | 389 | 0 | 0 | 215 | 244 | 2523 | **116** | 494 | 0 | 201 | 0 | 0 |
| 122 Sales, market research and development managers | 4897 | 0 | 0 | 2544 | 0 | 0 | 0 | 607 | 0 | 158 | 580 | 0 | 0 | 317 | 0 | 0 | **87** | 338 | 266 | 0 | 0 | 0 |
| 131 Production managers in farming, forestry and fishing | 134 | 134 | 0 | 0 | 0 | 0 | 0 | 0 | 0 | 0 | 0 | 0 | 0 | 0 | 0 | 0 | **0** | 0 | 0 | 0 | 0 | 0 |
| 132 Managers in processing, mining, constructions and distribution | 13835 | 0 | 0 | 8212 | 416 | 0 | 2704 | 1004 | 1339 | 0 | 160 | 0 | 0 | 0 | 0 | 0 | **0** | 0 | 0 | 0 | 0 | 0 |
| 133 Service managers in information and communication technologies | 1022 | 0 | 0 | 0 | 0 | 0 | 0 | 0 | 0 | 0 | 1022 | 0 | 0 | 0 | 0 | 0 | **0** | 0 | 0 | 0 | 0 | 0 |
| 134 Professional service managers | 15016 | 0 | 0 | 317 | 0 | 0 | 0 | 520 | 329 | 0 | 509 | 3003 | 272 | 0 | 306 | 949 | **5491** | 2328 | 549 | 444 | 0 | 0 |
| 141 Hotel and restaurant managers | 26370 | 0 | 0 | 0 | 0 | 0 | 0 | 0 | 0 | 26370 | 0 | 0 | 0 | 0 | 0 | 0 | **0** | 0 | 0 | 0 | 0 | 0 |
| 142 Managers in retail and wholesale trading | 25043 | 0 | 0 | 0 | 0 | 0 | 0 | 25043 | 0 | 0 | 0 | 0 | 0 | 0 | 0 | 0 | **0** | 0 | 0 | 0 | 0 | 0 |
| 143 Managers in other services | 7778 | 0 | 0 | 0 | 323 | 0 | 0 | 252 | 1753 | 97 | 904 | 0 | 0 | 122 | 2887 | 217 | **280** | 0 | 562 | 380 | 0 | 0 |
| 211 Professionals in the physical sciences and earth sciences | 7164 | 0 | 0 | 3135 | 0 | 322 | 0 | 404 | 0 | 0 | 0 | 219 | 0 | 2114 | 296 | 325 | **350** | 0 | 0 | 0 | 0 | 0 |
| 212 Mathematicians, actuaries and statisticians | 2744 | 0 | 0 | 578 | 0 | 0 | 0 | 0 | 0 | 0 | 0 | 356 | 0 | 501 | 180 | 225 | **521** | 0 | 0 | 384 | 0 | 0 |
| 213 Professional in the life sciences | 13459 | 883 | 0 | 740 | 0 | 0 | 0 | 3189 | 0 | 0 | 0 | 0 | 0 | 2351 | 430 | 3781 | **186** | 1362 | 537 | 0 | 0 | 0 |
| 214 Mechanics (excepting electro-technicians) | 57789 | 145 | 0 | 6473 | 1169 | 0 | 8326 | 822 | 2776 | 123 | 265 | 549 | 0 | 28504 | 0 | 7103 | **457** | 112 | 804 | 160 | 0 | 0 |
| 215 Electro-technicians | 12594 | 0 | 148 | 1268 | 835 | 0 | 438 | 721 | 0 | 0 | 3335 | 0 | 0 | 5034 | 0 | 472 | **0** | 128 | 216 | 0 | 0 | 0 |
| 216 Architects, surveyors, urban planners and designers | 24374 | 0 | 0 | 1003 | 469 | 160 | 1641 | 180 | 241 | 0 | 1574 | 0 | 398 | 13419 | 129 | 4367 | **244** | 0 | 550 | 0 | 0 | 0 |
| 221 Doctors in general | 51057 | 0 | 0 | 0 | 0 | 0 | 0 | 178 | 0 | 0 | 0 | 188 | 0 | 0 | 0 | 2040 | **254** | 48396 | 0 | 0 | 0 | 0 |
| 222 Nursing staff and midwives | 14254 | 0 | 0 | 0 | 0 | 0 | 0 | 0 | 0 | 354 | 0 | 0 | 0 | 160 | 0 | 773 | **268** | 12699 | 0 | 0 | 0 | 0 |
| 223 Practitioners of traditional and alternative medical professions | 0 | 0 | 0 | 0 | 0 | 0 | 0 | 0 | 0 | 0 | 0 | 0 | 0 | 0 | 0 | 0 | **0** | 0 | 0 | 0 | 0 | 0 |
| 224 Practitioners of paramedical professions | 1317 | 0 | 0 | 0 | 0 | 0 | 0 | 0 | 0 | 0 | 0 | 0 | 0 | 0 | 0 | 0 | **0** | 1317 | 0 | 0 | 0 | 0 |
| 225 Veterinary doctors | 2519 | 0 | 0 | 0 | 0 | 0 | 0 | 0 | 0 | 0 | 0 | 0 | 0 | 2272 | 0 | 160 | **0** | 86 | 0 | 0 | 0 | 0 |
| 226 Other professionals in health care services | 40247 | 0 | 0 | 758 | 0 | 0 | 0 | 13006 | 140 | 0 | 193 | 0 | 0 | 0 | 0 | 1029 | **175** | 24307 | 227 | 411 | 0 | 0 |
| 231 Teaching staff in tertiary education | 13761 | 0 | 0 | 0 | 0 | 0 | 0 | 0 | 0 | 0 | 0 | 0 | 0 | 0 | 0 | 0 | **13761** | 0 | 0 | 0 | 0 | 0 |
| 232 Teaching staff in vocational training | 11018 | 0 | 0 | 0 | 0 | 0 | 0 | 0 | 0 | 0 | 0 | 0 | 0 | 0 | 0 | 0 | **11018** | 0 | 0 | 0 | 0 | 0 |
| 233 Teaching staff in secondary education | 81203 | 0 | 0 | 0 | 0 | 0 | 0 | 0 | 0 | 0 | 0 | 0 | 0 | 0 | 0 | 0 | **81203** | 0 | 0 | 0 | 0 | 0 |
| 234 Teachers in primary education and kindergartens | 84120 | 0 | 0 | 0 | 0 | 0 | 0 | 0 | 0 | 0 | 0 | 0 | 0 | 0 | 0 | 0 | **83745** | 374 | 0 | 0 | 0 | 0 |
| 235 Other teaching staff | 61171 | 0 | 0 | 0 | 0 | 0 | 0 | 0 | 0 | 0 | 0 | 0 | 0 | 0 | 0 | 265 | **60906** | 0 | 0 | 0 | 0 | 0 |
| 241 Professionals in the fiscal sector | 78597 | 0 | 0 | 8007 | 1503 | 0 | 1665 | 10696 | 2131 | 1110 | 3635 | 3340 | 0 | 39344 | 922 | 3668 | **809** | 1030 | 0 | 738 | 0 | 0 |
| 242 Professionals in administration | 19181 | 0 | 0 | 1602 | 389 | 0 | 0 | 1082 | 549 | 121 | 2398 | 3230 | 0 | 1669 | 678 | 6242 | **793** | 170 | 0 | 259 | 0 | 0 |
| 243 Professionals in sales, market research and public relations | 17129 | 0 | 0 | 5642 | 0 | 0 | 258 | 4103 | 0 | 185 | 1875 | 223 | 0 | 3525 | 0 | 342 | **0** | 580 | 0 | 395 | 0 | 0 |
| 251 Designers and analysts of software and applications | 26304 | 0 | 0 | 1468 | 796 | 0 | 0 | 1796 | 107 | 0 | 16901 | 653 | 0 | 1266 | 0 | 1629 | **477** | 426 | 615 | 170 | 0 | 0 |
| 252 Data base and network professionals | 2139 | 0 | 0 | 0 | 0 | 0 | 0 | 0 | 0 | 0 | 1269 | 0 | 0 | 361 | 0 | 225 | **284** | 0 | 0 | 0 | 0 | 0 |
| 261 Professionals in law in general | 54323 | 0 | 0 | 403 | 279 | 0 | 464 | 0 | 0 | 0 | 434 | 709 | 0 | 44177 | 0 | 7397 | **0** | 0 | 0 | 134 | 0 | 325 |
| 262 Librarians, archivists and curators of antiquities, museums and art galleries | 1255 | 0 | 0 | 0 | 0 | 0 | 0 | 0 | 0 | 0 | 0 | 0 | 0 | 0 | 0 | 824 | **268** | 0 | 163 | 0 | 0 | 0 |
| 263 Professionals in the social and religious sector | 35489 | 0 | 0 | 709 | 441 | 0 | 0 | 166 | 483 | 631 | 277 | 1561 | 0 | 2933 | 306 | 4353 | **1943** | 9579 | 1009 | 10658 | 0 | 441 |
| 264 Writers, reporters and language professionals | 14799 | 0 | 0 | 364 | 0 | 0 | 0 | 0 | 0 | 0 | 10832 | 259 | 0 | 1868 | 102 | 158 | **576** | 0 | 368 | 273 | 0 | 0 |
| 265 Artists in general | 12603 | 0 | 0 | 0 | 0 | 0 | 0 | 0 | 0 | 2699 | 932 | 0 | 0 | 280 | 0 | 748 | **547** | 0 | 6145 | 1253 | 0 | 0 |
| 311 Technological staff in physics and engineering | 21465 | 162 | 143 | 3485 | 1612 | 625 | 3297 | 702 | 2401 | 0 | 1075 | 0 | 0 | 5380 | 0 | 1626 | **369** | 500 | 0 | 86 | 0 | 0 |
| 312 Supervisors in mining, processing and construction | 3167 | 0 | 313 | 551 | 0 | 202 | 1383 | 0 | 149 | 0 | 0 | 0 | 0 | 443 | 0 | 126 | **0** | 0 | 0 | 0 | 0 | 0 |
| 313 Technological staff in process control | 3542 | 0 | 159 | 1626 | 682 | 0 | 280 | 288 | 0 | 0 | 0 | 0 | 0 | 0 | 0 | 507 | **0** | 0 | 0 | 0 | 0 | 0 |
| 314 Technological staff in life sciences and related professions | 1896 | 171 | 0 | 106 | 0 | 274 | 0 | 132 | 0 | 0 | 0 | 0 | 0 | 363 | 0 | 0 | **0** | 850 | 0 | 0 | 0 | 0 |
| 315 Controllers and technicians of ships and aircraft | 14886 | 0 | 0 | 0 | 191 | 0 | 0 | 182 | 13470 | 0 | 0 | 0 | 0 | 0 | 249 | 550 | **0** | 244 | 0 | 0 | 0 | 0 |
| 321 Technical assistants of doctors and pharmacists | 15927 | 0 | 0 | 3188 | 0 | 0 | 0 | 1844 | 0 | 0 | 0 | 0 | 0 | 0 | 0 | 414 | **0** | 10481 | 0 | 0 | 0 | 0 |
| 322 Technological staff in nursing and obstetrics | 43615 | 0 | 0 | 132 | 0 | 0 | 190 | 0 | 0 | 0 | 0 | 540 | 0 | 0 | 0 | 999 | **0** | 41754 | 0 | 0 | 0 | 0 |
| 323 Technological staff in traditional and alternative medicine | 187 | 0 | 0 | 0 | 0 | 0 | 0 | 0 | 0 | 0 | 0 | 0 | 0 | 0 | 0 | 0 | **0** | 187 | 0 | 0 | 0 | 0 |
| 324 Technological staff and assistants of veterinary doctors | 819 | 0 | 0 | 0 | 0 | 0 | 0 | 271 | 0 | 0 | 0 | 0 | 0 | 251 | 0 | 80 | **0** | 216 | 0 | 0 | 0 | 0 |
| 325 Other technological staff in the health sector | 16088 | 0 | 0 | 246 | 0 | 0 | 0 | 1270 | 0 | 177 | 0 | 0 | 0 | 270 | 0 | 973 | **211** | 12715 | 0 | 224 | 0 | 0 |
| 331 Professionals in the fiscal and mathematical sector | 43895 | 117 | 0 | 3012 | 186 | 791 | 250 | 1269 | 523 | 1325 | 1326 | 17804 | 0 | 10518 | 0 | 4509 | **752** | 860 | 0 | 401 | 0 | 251 |
| 332 Real estate agents | 22830 | 0 | 0 | 870 | 0 | 0 | 0 | 2475 | 0 | 182 | 0 | 16578 | 859 | 324 | 773 | 0 | **0** | 212 | 557 | 0 | 0 | 0 |
| 333 Agents in service provision to companies | 10587 | 0 | 0 | 0 | 0 | 0 | 0 | 0 | 3224 | 199 | 0 | 204 | 2267 | 1472 | 2755 | 195 | **0** | 0 | 271 | 0 | 0 | 0 |
| 334 Administrative secretaries and specialized secretaries | 36537 | 165 | 0 | 3156 | 722 | 487 | 0 | 3238 | 1199 | 1767 | 1255 | 1116 | 0 | 4478 | 524 | 6446 | **2212** | 9130 | 270 | 372 | 0 | 0 |
| 335 Professionals in law enforcement | 10491 | 0 | 0 | 0 | 0 | 0 | 0 | 0 | 247 | 0 | 0 | 0 | 0 | 0 | 139 | 9798 | **159** | 0 | 147 | 0 | 0 | 0 |
| 341 Professionals in the legal, social or religious sector | 5096 | 0 | 0 | 0 | 0 | 0 | 0 | 0 | 0 | 0 | 0 | 148 | 0 | 1385 | 0 | 3366 | **198** | 0 | 0 | 0 | 0 | 0 |
| 342 Professionals in sports and physical education | 15707 | 0 | 0 | 0 | 0 | 0 | 0 | 111 | 0 | 382 | 0 | 0 | 0 | 0 | 0 | 250 | **373** | 450 | 12029 | 2112 | 0 | 0 |
| 343 Professionals in the arts and culture, and chefs | 11326 | 0 | 0 | 650 | 250 | 0 | 0 | 526 | 196 | 1207 | 0 | 0 | 0 | 6998 | 0 | 0 | **0** | 150 | 623 | 725 | 0 | 0 |
| 351 Operation technicians and user assistants in information and communication technologies | 22580 | 384 | 0 | 1866 | 0 | 0 | 0 | 1578 | 1235 | 389 | 11524 | 1025 | 0 | 1331 | 0 | 1096 | **0** | 829 | 0 | 1324 | 0 | 0 |
| 352 Technicians in radio communications and radio-television | 3518 | 0 | 0 | 0 | 0 | 0 | 0 | 288 | 0 | 0 | 2062 | 0 | 0 | 0 | 357 | 184 | **0** | 0 | 627 | 0 | 0 | 0 |
| 411 General task clerks | 182048 | 1537 | 139 | 12470 | 5835 | 2367 | 3027 | 24415 | 13704 | 1425 | 6987 | 11142 | 244 | 12607 | 5450 | 58355 | **8272** | 9551 | 2276 | 2112 | 0 | 132 |
| 412 Secretaries (general tasks) | 39763 | 312 | 0 | 1736 | 0 | 470 | 1755 | 3649 | 1314 | 334 | 2175 | 1777 | 228 | 4546 | 2052 | 5416 | **6878** | 4243 | 1377 | 1402 | 0 | 99 |
| 413 Keyboardists | 5827 | 0 | 0 | 427 | 259 | 0 | 0 | 1590 | 118 | 0 | 1030 | 692 | 418 | 223 | 604 | 0 | **152** | 315 | 0 | 0 | 0 | 0 |
| 421 Cashiers, tellers and related professions | 35944 | 0 | 0 | 174 | 1623 | 749 | 418 | 9646 | 1467 | 1576 | 317 | 10530 | 254 | 379 | 0 | 1016 | **0** | 106 | 7500 | 188 | 0 | 0 |
| 422 Customer information staff | 62604 | 0 | 0 | 1387 | 554 | 0 | 0 | 2130 | 3117 | 26458 | 8974 | 896 | 0 | 3584 | 8873 | 2140 | **0** | 3006 | 953 | 531 | 0 | 0 |
| 431 Staff in accounting offices and related professions | 28690 | 123 | 0 | 2360 | 342 | 261 | 1200 | 3871 | 1201 | 734 | 1023 | 4411 | 0 | 5439 | 841 | 5648 | **232** | 450 | 0 | 557 | 0 | 0 |
| 432 Staff for the registering of materials and transport services | 38106 | 156 | 411 | 5827 | 330 | 0 | 361 | 23476 | 4337 | 647 | 0 | 424 | 0 | 0 | 0 | 1150 | **147** | 405 | 225 | 208 | 0 | 0 |
| 441 Other office staff | 26367 | 0 | 0 | 1959 | 1506 | 0 | 0 | 2291 | 7502 | 162 | 279 | 360 | 0 | 1129 | 1248 | 7756 | **910** | 303 | 756 | 206 | 0 | 0 |
| 511 Travel companions, ticket inspectors and tourist guides | 7183 | 116 | 0 | 0 | 0 | 0 | 0 | 159 | 4495 | 265 | 0 | 0 | 0 | 0 | 1046 | 0 | **684** | 0 | 223 | 0 | 0 | 194 |
| 512 Cooks | 57930 | 82 | 0 | 334 | 0 | 0 | 0 | 516 | 272 | 52896 | 0 | 0 | 0 | 0 | 0 | 392 | **174** | 3100 | 0 | 164 | 0 | 0 |
| 513 Waiters, in general | 165531 | 0 | 0 | 507 | 0 | 0 | 0 | 969 | 0 | 159614 | 0 | 0 | 0 | 0 | 0 | 289 | **70** | 1666 | 2268 | 0 | 149 | 0 |
| 514 Hairdressers, beauticians and related professions | 40515 | 0 | 0 | 0 | 0 | 0 | 0 | 398 | 0 | 361 | 0 | 0 | 0 | 0 | 0 | 119 | **0** | 1415 | 0 | 37559 | 662 | 0 |
| 515 Housekeepers and building managers | 10038 | 185 | 179 | 518 | 0 | 0 | 0 | 95 | 0 | 4737 | 0 | 0 | 0 | 0 | 198 | 779 | **914** | 524 | 251 | 954 | 705 | 0 |
| 516 Other staff in the provision of personal services | 7827 | 0 | 0 | 160 | 0 | 0 | 0 | 0 | 0 | 0 | 447 | 0 | 0 | 0 | 0 | 311 | **4046** | 721 | 0 | 1172 | 969 | 0 |
| 521 Traveling salespeople and open-air market salesmen | 21544 | 0 | 0 | 682 | 0 | 0 | 0 | 20703 | 0 | 159 | 0 | 0 | 0 | 0 | 0 | 0 | **0** | 0 | 0 | 0 | 0 | 0 |
| 522 Salespeople in shops | 403107 | 431 | 0 | 20868 | 212 | 0 | 901 | 363351 | 1098 | 8383 | 2787 | 0 | 0 | 240 | 3227 | 81 | **0** | 240 | 676 | 610 | 0 | 0 |
| 523 Cashiers and staff selling tickets | 26998 | 0 | 0 | 0 | 262 | 0 | 141 | 19221 | 1678 | 2128 | 0 | 2649 | 0 | 0 | 508 | 0 | **0** | 186 | 226 | 0 | 0 | 0 |
| 524 Other sales staff | 30615 | 0 | 0 | 1736 | 0 | 0 | 0 | 25972 | 0 | 1957 | 745 | 0 | 0 | 0 | 206 | 0 | **0** | 0 | 0 | 0 | 0 | 0 |
| 531 Childcare providers and teacher assistants | 21858 | 0 | 0 | 138 | 0 | 0 | 0 | 0 | 136 | 168 | 0 | 0 | 0 | 136 | 0 | 56 | **2604** | 12800 | 896 | 0 | 4923 | 0 |
| 532 Staff providing personal care in the sector of health services | 13986 | 0 | 0 | 360 | 0 | 0 | 0 | 1241 | 0 | 0 | 0 | 0 | 0 | 0 | 0 | 796 | **534** | 10403 | 0 | 0 | 651 | 0 |
| 541 Staff in the provision of security services | 101766 | 1207 | 0 | 1160 | 357 | 329 | 278 | 384 | 2096 | 717 | 690 | 257 | 0 | 0 | 19582 | 70497 | **608** | 0 | 3130 | 126 | 0 | 350 |
| 611 Farmers marketing their produce | 330327 | 326816 | 0 | 341 | 0 | 0 | 0 | 0 | 0 | 570 | 168 | 0 | 0 | 0 | 1383 | 732 | **0** | 0 | 0 | 318 | 0 | 0 |
| 612 Livestock breeders | 53587 | 53017 | 0 | 570 | 0 | 0 | 0 | 0 | 0 | 0 | 0 | 0 | 0 | 0 | 0 | 0 | **0** | 0 | 0 | 0 | 0 | 0 |
| 613 Farmers and stockbreeders with mixed farming | 43565 | 43565 | 0 | 0 | 0 | 0 | 0 | 0 | 0 | 0 | 0 | 0 | 0 | 0 | 0 | 0 | **0** | 0 | 0 | 0 | 0 | 0 |
| 621 Foresters, loggers and related professions | 2360 | 2360 | 0 | 0 | 0 | 0 | 0 | 0 | 0 | 0 | 0 | 0 | 0 | 0 | 0 | 0 | **0** | 0 | 0 | 0 | 0 | 0 |
| 622 Fishermen, hunters and wildlife trappers | 8161 | 8161 | 0 | 0 | 0 | 0 | 0 | 0 | 0 | 0 | 0 | 0 | 0 | 0 | 0 | 0 | **0** | 0 | 0 | 0 | 0 | 0 |
| 631 Farmers for private consumption | 0 | 0 | 0 | 0 | 0 | 0 | 0 | 0 | 0 | 0 | 0 | 0 | 0 | 0 | 0 | 0 | **0** | 0 | 0 | 0 | 0 | 0 |
| 632 Stockbreeders for private consumption | 0 | 0 | 0 | 0 | 0 | 0 | 0 | 0 | 0 | 0 | 0 | 0 | 0 | 0 | 0 | 0 | **0** | 0 | 0 | 0 | 0 | 0 |
| 633 Mixed farming and stockbreeding for private consumption | 0 | 0 | 0 | 0 | 0 | 0 | 0 | 0 | 0 | 0 | 0 | 0 | 0 | 0 | 0 | 0 | **0** | 0 | 0 | 0 | 0 | 0 |
| 634 Fishermen, hunters and wildlife trappers for private consumption | 0 | 0 | 0 | 0 | 0 | 0 | 0 | 0 | 0 | 0 | 0 | 0 | 0 | 0 | 0 | 0 | **0** | 0 | 0 | 0 | 0 | 0 |
| 711 Builders and related professions | 42409 | 159 | 85 | 5476 | 0 | 0 | 35092 | 173 | 0 | 1013 | 0 | 0 | 0 | 171 | 0 | 0 | **0** | 0 | 239 | 0 | 0 | 0 |
| 712 Technicians in the completion of building and related professions | 39830 | 0 | 132 | 2522 | 978 | 836 | 26532 | 3539 | 312 | 959 | 0 | 0 | 0 | 171 | 0 | 2579 | **0** | 596 | 450 | 223 | 0 | 0 |
| 713 House painters, dyers, cleaners and related professions | 15784 | 0 | 0 | 1079 | 0 | 0 | 12596 | 1259 | 0 | 181 | 0 | 0 | 0 | 0 | 353 | 315 | **0** | 0 | 0 | 0 | 0 | 0 |
| 721 Metal casters, smelters, sheet metal workers etc | 16243 | 70 | 745 | 9430 | 715 | 0 | 1986 | 2845 | 0 | 203 | 0 | 0 | 0 | 0 | 0 | 0 | **0** | 0 | 248 | 0 | 0 | 0 |
| 722 Ironsmiths, tool makers and related professions | 14241 | 0 | 0 | 12706 | 0 | 0 | 595 | 940 | 0 | 0 | 0 | 0 | 0 | 0 | 0 | 0 | **0** | 0 | 0 | 0 | 0 | 0 |
| 723 Mechanics and repairmen of machinery | 53946 | 409 | 1005 | 9584 | 203 | 231 | 1301 | 33544 | 1982 | 260 | 187 | 94 | 0 | 505 | 196 | 3322 | **0** | 178 | 825 | 0 | 0 | 119 |
| 731 Handicraftsmen | 8703 | 0 | 0 | 6701 | 0 | 0 | 0 | 1468 | 0 | 0 | 0 | 0 | 0 | 162 | 0 | 0 | **0** | 0 | 0 | 372 | 0 | 0 |
| 732 Printers and related professions | 12810 | 0 | 0 | 10976 | 0 | 0 | 0 | 360 | 0 | 0 | 463 | 0 | 0 | 810 | 200 | 0 | **0** | 0 | 0 | 0 | 0 | 0 |
| 741 Installers and repairmen of electrical equipment | 51913 | 114 | 576 | 7257 | 3704 | 308 | 20625 | 8521 | 1023 | 2265 | 559 | 0 | 0 | 733 | 0 | 2674 | **0** | 945 | 175 | 2434 | 0 | 0 |
| 742 Installers and repairmen of electronic and telecommunications equipment | 11711 | 0 | 87 | 541 | 317 | 0 | 742 | 2417 | 0 | 0 | 4914 | 207 | 0 | 0 | 174 | 1005 | **0** | 247 | 0 | 1060 | 0 | 0 |
| 751 Technicians in food processing and related professions | 51069 | 162 | 0 | 38618 | 0 | 0 | 0 | 8663 | 0 | 3212 | 0 | 0 | 0 | 0 | 0 | 0 | **114** | 301 | 0 | 0 | 0 | 0 |
| 752 Technicians in wood processing, cabinetmakers and related professions | 12943 | 0 | 0 | 11856 | 0 | 0 | 1087 | 0 | 0 | 0 | 0 | 0 | 0 | 0 | 0 | 0 | **0** | 0 | 0 | 0 | 0 | 0 |
| 753 Technicians in clothing and related professions | 19060 | 0 | 0 | 15687 | 0 | 0 | 0 | 485 | 268 | 0 | 0 | 0 | 0 | 0 | 0 | 0 | **0** | 0 | 0 | 2619 | 0 | 0 |
| 754 Other technicians and related professions | 2032 | 127 | 152 | 594 | 0 | 0 | 227 | 0 | 102 | 0 | 0 | 0 | 0 | 0 | 0 | 705 | **126** | 0 | 0 | 0 | 0 | 0 |
| 811 Operators of ore mining and ore processing systems | 8274 | 0 | 4461 | 2518 | 665 | 0 | 630 | 0 | 0 | 0 | 0 | 0 | 0 | 0 | 0 | 0 | **0** | 0 | 0 | 0 | 0 | 0 |
| 812 Operators of equipment for the processing and finish of metal products | 2312 | 0 | 0 | 2098 | 0 | 0 | 0 | 0 | 214 | 0 | 0 | 0 | 0 | 0 | 0 | 0 | **0** | 0 | 0 | 0 | 0 | 0 |
| 813 Operators of facilities and machinery for the production of chemical and photographic products. | 6567 | 0 | 0 | 5845 | 0 | 0 | 0 | 0 | 0 | 0 | 0 | 233 | 0 | 229 | 260 | 0 | **0** | 0 | 0 | 0 | 0 | 0 |
| 814 Operators of equipment for the production of elastic, plastic, paper and cardboard items | 10097 | 0 | 0 | 9796 | 0 | 0 | 0 | 0 | 0 | 0 | 0 | 0 | 0 | 0 | 0 | 0 | **0** | 0 | 0 | 301 | 0 | 0 |
| 815 Operators of equipment for textile, leather and fur products | 7041 | 0 | 0 | 3298 | 0 | 0 | 0 | 0 | 0 | 170 | 0 | 0 | 0 | 0 | 198 | 0 | **213** | 126 | 0 | 3037 | 0 | 0 |
| 816 Operators of equipment for the production of food items and related products | 19569 | 162 | 0 | 18707 | 0 | 0 | 0 | 567 | 0 | 0 | 0 | 0 | 0 | 0 | 134 | 0 | **0** | 0 | 0 | 0 | 0 | 0 |
| 817 Operators of processing equipment for wood, paper and cardboard | 3056 | 0 | 0 | 2821 | 0 | 236 | 0 | 0 | 0 | 0 | 0 | 0 | 0 | 0 | 0 | 0 | **0** | 0 | 0 | 0 | 0 | 0 |
| 818 Other operators of stationary equipment and machinery for the production of paper products. | 3854 | 0 | 127 | 1977 | 356 | 562 | 0 | 182 | 0 | 0 | 0 | 0 | 0 | 0 | 417 | 0 | **0** | 0 | 233 | 0 | 0 | 0 |
| 821 Assemblers (fitters) | 3413 | 0 | 0 | 3146 | 0 | 0 | 0 | 0 | 0 | 0 | 267 | 0 | 0 | 0 | 0 | 0 | **0** | 0 | 0 | 0 | 0 | 0 |
| 831 Engine drivers and related professions | 265 | 0 | 0 | 0 | 0 | 0 | 105 | 0 | 160 | 0 | 0 | 0 | 0 | 0 | 0 | 0 | **0** | 0 | 0 | 0 | 0 | 0 |
| 832 Drivers of vehicles, small trucks and motorcycles | 80223 | 117 | 0 | 2100 | 779 | 593 | 338 | 6090 | 50569 | 14196 | 0 | 373 | 0 | 0 | 1389 | 2011 | **255** | 833 | 0 | 583 | 0 | 0 |
| 833 Drivers of trucks and buses | 83774 | 120 | 283 | 9036 | 308 | 5582 | 1998 | 15594 | 40341 | 0 | 194 | 0 | 0 | 0 | 3373 | 3049 | **1387** | 1755 | 0 | 754 | 0 | 0 |
| 834 Operators of movable equipment | 23995 | 479 | 1201 | 4308 | 228 | 630 | 9660 | 1376 | 2811 | 0 | 212 | 0 | 0 | 0 | 1185 | 1905 | **0** | 0 | 0 | 0 | 0 | 0 |
| 835 Maritime staff – deck crew and related professions | 4895 | 0 | 0 | 0 | 0 | 0 | 0 | 0 | 4643 | 0 | 0 | 0 | 0 | 0 | 0 | 0 | **251** | 0 | 0 | 0 | 0 | 0 |
| 911 Cleaners and assistants in homes, hotels and offices | 103439 | 0 | 0 | 3054 | 0 | 0 | 1046 | 2822 | 1112 | 30346 | 383 | 480 | 0 | 70 | 19002 | 4382 | **9330** | 7212 | 598 | 979 | 22623 | 0 |
| 912 Cleaners of vehicles, windows and related professions | 6057 | 0 | 0 | 610 | 0 | 0 | 0 | 2220 | 0 | 335 | 0 | 0 | 0 | 0 | 1156 | 185 | **160** | 247 | 0 | 1143 | 0 | 0 |
| 921 Workers in farming, forestry and fishing | 31173 | 28711 | 0 | 918 | 0 | 0 | 0 | 0 | 0 | 0 | 0 | 0 | 0 | 0 | 1425 | 119 | **0** | 0 | 0 | 0 | 0 | 0 |
| 931 Workers in mines and construction | 14158 | 0 | 534 | 2181 | 1532 | 447 | 6576 | 803 | 0 | 628 | 0 | 0 | 0 | 0 | 0 | 1062 | **0** | 0 | 394 | 0 | 0 | 0 |
| 932 Workers in processing | 35547 | 1215 | 163 | 29266 | 0 | 765 | 588 | 1397 | 0 | 0 | 0 | 0 | 0 | 0 | 1321 | 336 | **0** | 0 | 0 | 496 | 0 | 0 |
| 933 Workers in transport and storage | 16934 | 0 | 0 | 3436 | 158 | 0 | 0 | 8384 | 3675 | 542 | 402 | 0 | 0 | 0 | 177 | 0 | **0** | 0 | 0 | 160 | 0 | 0 |
| 941 Help in food production | 26403 | 0 | 0 | 587 | 0 | 0 | 0 | 0 | 0 | 24819 | 0 | 0 | 0 | 0 | 0 | 220 | **0** | 582 | 194 | 0 | 0 | 0 |
| 951 Persons offering minor services outdoors and related professions | 2444 | 0 | 0 | 0 | 0 | 0 | 0 | 509 | 0 | 297 | 266 | 0 | 0 | 1150 | 222 | 0 | **0** | 0 | 0 | 0 | 0 | 0 |
| 952 Street vendors (excepting food sales) | 727 | 0 | 0 | 0 | 0 | 0 | 0 | 502 | 0 | 0 | 0 | 0 | 0 | 0 | 0 | 0 | **0** | 0 | 224 | 0 | 0 | 0 |
| 961 Refuse collectors, in general | 18041 | 0 | 0 | 0 | 0 | 14249 | 0 | 0 | 0 | 0 | 0 | 0 | 0 | 0 | 722 | 2653 | **417** | 0 | 0 | 0 | 0 | 0 |
| 962 Other unspecialized workers, manual workers and small business operators | 14548 | 456 | 132 | 2028 | 639 | 566 | 604 | 2002 | 1010 | 2472 | 0 | 0 | 0 | 1088 | 282 | 1110 | **0** | 0 | 1250 | 816 | 94 | 0 |
| Ο11 Non-classifiable persons | 65192 | 0 | 0 | 0 | 0 | 0 | 0 | 0 | 0 | 0 | 0 | 0 | 0 | 0 | 0 | 65192 | **0** | 0 | 0 | 0 | 0 | 0 |
